# Wearable Devices for Assessment of Tremor

**DOI:** 10.3389/fneur.2021.680011

**Published:** 2021-06-11

**Authors:** Basilio Vescio, Andrea Quattrone, Rita Nisticò, Marianna Crasà, Aldo Quattrone

**Affiliations:** ^1^Biotecnomed S.C.aR.L., Catanzaro, Italy; ^2^Department of Medical and Surgical Sciences, Institute of Neurology, Magna Græcia University, Catanzaro, Italy; ^3^Neuroimaging Unit, Institute of Molecular Bioimaging and Physiology of the National Research Council (IBFM-CNR), Catanzaro, Italy; ^4^Department of Medical and Surgical Sciences, Neuroscience Research Center, Magna Græcia University, Catanzaro, Italy

**Keywords:** tremor, wearable devices, Parkinson's disease, essential tremor, monitoring, diagnosis

## Abstract

Tremor is an impairing symptom associated with several neurological diseases. Some of such diseases are neurodegenerative, and tremor characterization may be of help in differential diagnosis. To date, electromyography (EMG) is the gold standard for the analysis and diagnosis of tremors. In the last decade, however, several studies have been conducted for the validation of different techniques and new, non-invasive, portable, or even wearable devices have been recently proposed as complementary tools to EMG for a better characterization of tremors. Such devices have proven to be useful for monitoring the efficacy of therapies or even aiding in differential diagnosis. The aim of this review is to present systematically such new solutions, trying to highlight their potentialities and limitations, with a hint to future developments.

## Introduction

Tremor is generally defined as an involuntary, rhythmic, oscillatory movement of a body part (1). Limbs and head, when unsupported, may exhibit slight tremor, referred to as physiological tremor. Such tremor is generally not visible or symptomatic unless it is enhanced by fatigue or anxiety. Pathological tremor, on the other hand, is usually visible and persistent and can severely compromise the execution of normal life tasks, like eating, dressing, writing.

**Figure 1:**
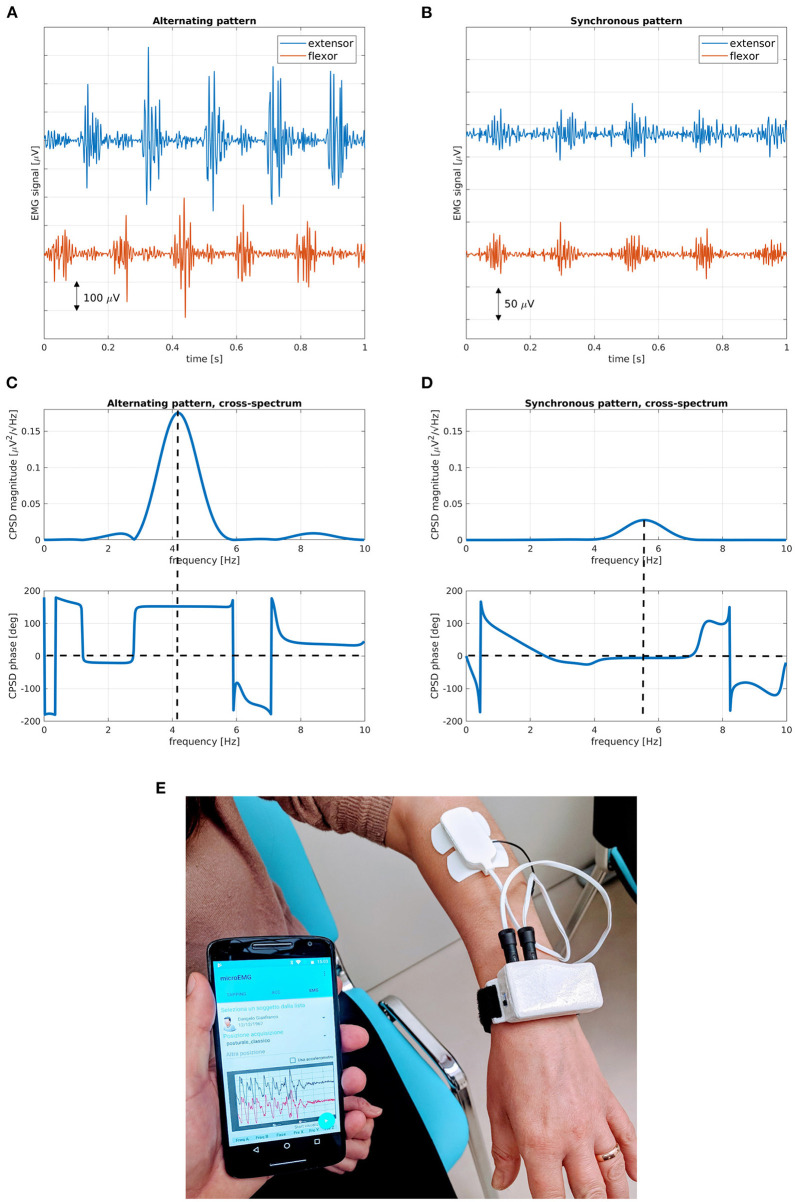
Electrophysiological and spectral characteristics of tremor patterns. Muscle bursts for **(A)** alternating and **(B)** synchronous tremor patterns; magnitude and phase cross-spectral diagrams for **(C)** alternating and **(D)** synchronous tremor patterns. **(E)** Wrist-worn device, with EMG plates and mobile app for the characterization of tremor patterns. Alternating bursts of antagonist muscles show a marked phase difference at peak tremor frequency, while synchronous bursts have a small phase difference at peak tremor frequency. In alternating tremors, peak amplitude is usually higher and average frequency is lower than in synchronous tremors. EMG, electromyography; CPSD, cross power spectral density.

Tremor symptoms may affect one body region (focal tremor), two or more adjacent parts (segmental tremor), one side (hemitremor), or the whole body (generalized tremor). According to activation conditions, two kinds of tremors are generally considered: *rest tremor*, when the affected part is relaxed, and *action tremor* (kinetic, postural, or isometric), when the subject performs voluntary movements or voluntarily maintains a certain position against gravity. Tremor features include frequency (usually in the range of 4–8 Hz) and amplitude. When two or more antagonist muscles are involved in tremor, activation patterns are defined according to the relative timing of tremor electromyography (EMG) bursts: synchronous pattern, when muscle bursts are in phase, and alternating pattern, when bursts are phase-shifted (2), as shown in Figures 1A–D.

Surface EMG is the gold standard technique for the diagnosis, characterization, and monitoring of tremor (3). Unfortunately, it suffers from uncertainty and errors due to bad positioning of electrodes, changes in skin conductance, and cross-talking from other muscles. To avoid such inconveniences, needle EMG (4) is the most reliable technique for a precise characterization of tremor features, but it is invasive and costly.

Generally, EMG is unsuitable for continuous monitoring or frequent assessment of tremor characteristics.

In the last decade, the large diffusion of mobile devices has fostered the development of several portable and wearable solutions for health monitoring or even for disease diagnosis. Most of such devices are based on inertial sensors (accelerometers and gyroscopes), while others use a combination of inertial and electrophysiological information. Many of them can be interfaced with smartphones or tablets through wireless communication protocols (Bluetooth, Wi-Fi, etc.). Smartphones, smartwatches, and tablets have sufficient computing resources for performing complex calculations, such as digital signal processing and artificial intelligence (AI).

Mobile devices, together with the advent of the Internet of Things (IoT), have dramatically changed people's lifestyles and have found newer and newer areas of application, allowing for continuous monitoring of disease symptoms and vital signs. However, signal processing techniques and sensing technologies need to be properly selected in order to provide data in agreement with the clinical-functional assessment of tremor (5).

In this brief review, we mainly focus on novel wearable solutions for the automated acquisition and analysis of tremor data. For this purpose, three main classes of wearable devices are identified: (1) devices for assessing tremor features, (2) devices for monitoring tremor and efficacy of therapies, and (3) devices for differential diagnosis between tremulous disorders. Table 1 reports a synthetic view and classification of the examined literature.

**Table 1:** Classification of examined literature.

Assessment of tremor features	Sensors on fingers/hand/wrist	• Elble (6) • Heldman (7) • Dai (8) • Marino (9) • Hssayeni (10)	• Mahadevan (11) • Dai (12) • Sanchez-Perez (13) • Jeon (14)
	Sensors on multiple segments/whole body	• Rigas (15) • Charles (16) • Lonini (17)	• Huo (18) • Delrobaei (19)
	Smartphone based methods	• LeMoyne (20) • LeMoyne (21)	• Araújo (22) • Bhatti (23)
	Smartwatch based methods	• López-Blanco (24) • Varghese (25)	• López-Blanco (26) • Shawen (27)
	Other devices	• Zajki-Zechmeister (28)	
Continuous monitoring of tremor		• Cole (29) • Jeonghee (30) • Battista (31) • Battista (32) • Heijmans (33)	• San-Segundo (34) • McNames (35) • Kuosmanen (36) • Erb (37)
Differential diagnosis between tremors		• Vescio (38) • Hossen (39) • Ghassemi (40)	• Di Biase (41) • Bove (42)

## Methods

For the purposes of this review, PubMed and Google Scholar search engines were queried using combinations of the following keywords: tremor, wearable, device, assessment, monitoring, and diagnosis. The words “tremor” and “wearable” were used as fixed keys in all searching queries. Only articles published in the last decade were selected.

## Devices for the Assessment and Characterization of Tremor

Inertial sensors have proven to be of great help in clinical practice (43), especially in the assessment, diagnosis, and treatment of tremor in Parkinson's disease (PD) (44–46).

The large diffusion of smartphones, tablets, and smartwatches has fostered the development of specialized software applications that make use of on-board sensors for inertial measurements of tremor and other movement alterations (20–23). LeMoyne et al. (20) used a common smartphone for estimating tremor frequency in PD subjects. The authors used the same equipment for assessing tremor in essential tremor (ET) subjects (21), discriminating between *on* and *off* state during deep brain stimulation (DBS). Araújo et al. (22) found a good agreement between EMG measurements and accelerometer estimations made by three different mobile apps. A similar approach was used by Bhatti et al. (23) for the evaluation of orthostatic tremor. However, these solutions can reliably estimate frequency only.

Along with the introduction of smartphone apps, several dedicated devices and methods have been proposed for tremor measurement. A summary of characteristics and specifications required for motion sensing transducers and analysis methods for assessing tremor severity in terms of amplitude and occurrence is reported by Elble and McNames (6).

Heldman et al. (7) evaluated a commercial motion-sensing device, worn on the hand or fingers of the most affected side, in ET subjects while performing motion tasks. The results of this study opened a way toward continuous rating of tremor severity during routine or spontaneous activities of daily living. Other hand- or wrist-wearable devices were introduced later for evaluating rest and action (postural and isometric) tremor in PD subjects using an inertial measurement unit (IMU), made of a triaxial accelerometer and a triaxial gyroscope, both on the same silicon chip (8), or a set of four triaxial accelerometers (9). An IMU was also used by Hssayeni et al. (10) to assess tremor severity in PD and by Mahadevan et al. (11) and Dai et al. (12) in order to discriminate between bradykinesia and tremor. Sanchez-Perez et al. (13) devised a novel algorithm based on fuzzy logic for the evaluation of rest tremor severity. These authors achieved a good level of agreement with Unified Parkinson Disease Rating Scale (UPDRS) part III (10–13), thus showing the equivalence between clinical scales and tremor assessment by wearable sensors.

A wrist-worn device with and external IMU placed on a finger was proposed by Jeon et al. (14), together with various AI techniques for the automatic scoring of rest tremor in PD. Other studies (24–27) have focused on the use of commercial smartwatches, which have become easily available in the last years. López-Blanco et al. were able to correlate the root mean square of angular velocity acquired from the triaxial gyroscope of an Android-based smartwatch to the Fahn–Tolosa–Marin (FTM) tremor rating scale (TRS) scores of ET subjects (24) and to UPDRS-III scores of PD subjects (26). Varghese et al. (25) used a smartwatch within an integrated analysis framework comprising a smartphone and a tablet for the implementation of a tremor assessment and monitoring system in a clinical setting. Shawen et al. (27) compared the performances of a smartwatch and a skin-mounted IMU in classifying tremor and bradykinesia severity in PD, demonstrating that smartwatch performance was comparable to that of a custom, specialized sensor.

By extending such localized measurement systems to a distributed configuration, other solutions have been devised, including more sensors displaced on several body points or limbs. Rigas et al. (15) developed a method based on features extracted from accelerometers mounted in different body segments, which produce data feeding two parallel hidden Markov models (HMM): the first one is used to quantify tremor severity and the second one to recognize body posture and action, thus providing a complete assessment of tremor activity. A preliminary study (16) used three electromagnetic motion capture sensors on different limbs of the arm. The aim of this study was to provide a model for tremor-suppression orthotic strategies in ET, but no progressions have been made so far in such direction. A more complex setup was proposed in a study by Lonini et al. (17), where PD subjects where instrumented with six multi-modal soft sensors (triaxial accelerometers and gyroscopes, with two-lead skin surface voltage), capable of acquiring accelerations, angular velocity, and EMG while deforming with skin. This setup was used to assess the performances of AI models in detecting motor symptoms (tremor and bradykinesia) during normal life activities. Huo et al. (18) introduced an even more complex suit, based on a force sensor, three IMUs, and four custom mechanomyography (MMG) sensors. The system was tested in its capacity to predict Unified Parkinson's Disease Rating Scale (UPDRS) scores based on quantitative assessment of bradykinesia, rigidity, and tremor in PD patients. Delrobaei et al. (19) performed a similar task using a distributed setup with 17 wireless IMUs, hinting at possible applications in home-monitoring settings. Another system, in the form of a pen, has been described by Zajki-Zechmeister et al. (28) and can provide information comparable to tremor scales, MDS-UPDRS for PD, and Essential Tremor Rating Assessment Scale (TETRAS) for ET. Despite the large diffusion of wearable sensors for the assessment of tremor features and for the evaluation of tremor severity, these technologies are still rarely used in clinical practice. It has been demonstrated that their evaluation of tremor severity and their test–retest variability are comparable to those of rating scales (6). These wearable solutions can reliably estimate only tremor frequency and amplitude and can be used as the basis for the development of more complex devices for the differential diagnosis of tremulous disorders and for the monitoring of therapies.

## Devices for Monitoring Tremor and Efficacy of Therapies

Continuous monitoring of tremor symptoms has gained an increasing interest in the last years due to the continuous need for home-care solutions and smart services capable of reducing the burden of National Health Systems. Monitoring tremors during normal life activities can help in assessing the efficacy of therapies. It may be useful for understanding when tremor occurs and whether it is related to specific tasks or conditions. The main difficulty in daily life tracking is the reliable discrimination of tremor from other movements and artifacts. Therefore, a great effort has been dedicated to the development of signal processing and AI techniques.

Cole et al. (29) validated a network of eight wireless sensors with combined 3D accelerometry and surface EMG and tested several machine learning (ML) algorithms for the assessment of the presence/absence and severity of tremor and dyskinesia. They proved that their strategy achieved a small error rate and was robust to changes in the positioning of sensors. Kim et al. (30) used a wrist-worn device equipped with an IMU and statistical pattern recognition algorithms to discriminate upper limbs tremor from normal daily activities. Another watch-like device, based on a triaxial accelerometer, was introduced and validated by Battista and Romaniello (31, 32). Their device was used to identify tremor events by computing statistical indexes that were representative of motion patterns. In addition to a wrist IMU sensor, Heijmans et al. (33) used also a second IMU positioned on the chest, together with a questionnaire for annotating tremor events during the day. The annotated data were used to predict tremor severity. A wrist-worn accelerometer, together with a smartphone annotation app, was used by San-Segundo et al. (34). In this study, labeled data were collected in a laboratory setting and weak-labeled data were recorded during daily life. Several AI models were used to identify tremor occurrence and severity from different sets of extracted features.

McNames et al. (35) use two IMUs, one for each wrist, and a two-stage algorithm for refining tremor frequency estimation during the normal activity of PD subjects for seven consecutive days. A smartphone-based solution for long-term monitoring was introduced by Kuosmanen et al. (36), consisting of an accelerometer-based ball game for quantifying patients' hand tremor, a medication journal for logging medication intake times, a daily survey for reporting the overall severity of PD symptoms, and reminder notifications. Erb et al. (37) introduced four different studies based on home monitoring by means of wearable sensors and self-reporting diaries. In this work, several sensing technologies were used: accelerometers, gyroscopes, magnetometers, barometers, electrocardiogram (ECG), EMG, and galvanic skin response (GSR) sensors. The main limitation of the proposed solutions is the accuracy in distinguishing between tremor and other movements or artifacts, due to the high variability of signals recorded during normal daily activity. Such monitoring devices seem to work better in combination with self-annotations. Achieving a good accuracy in identifying tremor and in assessing its severity during continuous, fully automated monitoring is still an open challenge.

## Devices for Differential Diagnosis

Differential diagnosis between tremulous disorders is, perhaps, one of the most intriguing and challenging research tasks that have been carried on in recent times. A successful discrimination between neurological diseases based on tremor data only may avoid more complex, invasive, and expensive examinations. Hence, the interest for simpler instruments and methods may help even general practitioners in screening neurological disorders that exhibit tremor symptoms. Discrimination of ET from PD and other neurodegenerations often requires a DAT-SPECT imaging examination. Such examination is costly and invasive, as it employs a radioactive tracer. Essential tremor subjects have normal DAT-SPECT; therefore, abnormal DAT-SPECT can be considered as an exclusion criterion for ET (47). The increasing availability of cheap, non-invasive sensors and the development of ML and signal processing techniques have supported the search for alternative biomarkers in the huge amount of data that can be easily produced.

Nisticò et al. first discovered the usefulness of phase pattern in antagonistic muscle pairs as a powerful biomarker capable of discriminating ET from PD (48) and drug-induced Parkinsonism (DIP) from PD (49). Their work was based on EMG recordings and automatic evaluation of phase lags between bursts detected on the extensor carpi radialis (ECR) and flexor carpi ulnaris (FCU) muscles during rest tremor occurrence. It was observed that PD subjects exhibited an alternating activation pattern, with a marked phase shift between bursts corresponding to the alternating contractions of the antagonistic muscle pair. Non-PD subjects (ET and DIP) exhibited synchronous patterns, with no significant phase shift and muscles contracting at the same time. These findings have led to the development and validation of a wearable watch-like device (38), equipped with two EMG acquisition plates (one for each muscle) and with wireless connection to a smartphone and mobile app for real-time processing and fully automated evaluation (Figure 1E). The system is capable of characterizing rest tremor phase pattern in <1 min and to discriminate between PD and non-PD on an individual basis.

Other authors (39, 40) introduced AI-based analysis techniques for discriminating ET from PD using combined EMG and accelerometer signals acquired in a laboratory setting. Overall discrimination accuracies were 88.75 and 83%, respectively. However, such methods have not been implemented in any device yet.

Di Biase et al. (41) introduced another biomarker, called tremor stability index (TSI), evaluated by means of a triaxial accelerometer mounted on the wrist. Tremor stability index is evaluated as the interquartile range of the instantaneous frequency change. The authors tested this index on different datasets, achieving an accuracy between 82% (on a validation cohort) and 90% (testing cohort) in discriminating ET from PD. Bove et al. (42) used triaxial accelerometers worn on the proximal one-third of the metacarpals, and evaluated differences in frequency, amplitude, coherence, and peak dispersion of resting and action tremor between PD, ET, and dystonic tremor (DT) subjects. They combined these parameters into three sets of at most five discriminating criteria (one set for each disease), achieving, respectively, the following values of sensitivity and specificity: for DT, 85 and 87.5%; for ET, 95 and 90%; for PD, 100 and 93%. Diagnostic solutions based on inertial sensors have achieved a good discriminating performance. Wearable EMG devices, however, show the best accuracy in differential diagnosis between tremulous disorders, as they can evaluate tremor patterns.

## Conclusion

Wearable sensors have undergone important developments in the last decade in an increasing number of areas of application. Healthcare is one of the most promising sectors, where new technologies are being used for sensing, acquiring, analyzing, and sharing data. Several wearable solutions have been implemented, either using commercially available devices or developing custom systems, for aiding clinical evaluation and diagnosis. In this short review, we have focused on devices and solutions for the assessment, continuous monitoring, and diagnosis of tremor in neurological diseases. As a first consideration, up to date, most wearable applications are mainly focused on tremor assessment and quantification of tremor severity. A minor number of solutions are dedicated to home monitoring of tremor symptoms in order to fully characterize their occurrence and severity during daily life tasks and to optimize therapies. The use of wearable technologies for differential diagnosis between tremulous disorders is very promising. In the next future, more efforts will be devoted to this field. Another consideration regards sensing technologies. Inertial sensing based on Micro Electro-Mechanical Systems (MEMS) is still the most used technology for wearable devices measuring tremor. This is mainly due to their physical properties: tremor is a rhythmic movement, and these transducers sense motion. Moreover, they are nearly ubiquitous, as they are embedded in all mobile communication and entertainment devices, in smartwatches and smart bands used for sports and fitness. Last, they can be easily embedded in any wearable solution thanks to their small dimensions and low power requirements. However, in diagnostic applications, the accuracy that can be achieved using MEMS sensors is still lower than that of solutions that include EMG and tremor pattern analysis.

## Future Perspectives

The pervasive diffusion of mobile devices and network services, together with the advancement of signal processing algorithms, will allow for a wider diffusion of wearable solutions for diagnosing and monitoring tremors and other pathological conditions. Skin sensors, which can be used as patches, represent another emerging technology. They are at a very early stage but are very likely to be used in the next future for continuous monitoring applications. New devices will mainly follow three development directions: (i) smaller sizes, (ii) more complex and intelligent processing algorithms, and (iii) wireless interconnection to other devices and to more and more complex services on the Internet. The combination of these characteristics will allow for the development of new sophisticated devices for diagnostic and monitoring applications.

## Author Contributions

AlQ and RN: conception of the work. BV, AnQ, and MC: literature review. BV: first draft of the manuscript. AnQ, RN, and MC: contribution to the writing of all sections. AlQ and RN: critical review of the manuscript. All authors contributed to manuscript revision and read and approved the submitted version.

## Conflict of Interest

The authors declare that the research was conducted in the absence of any commercial or financial relationships that could be construed as a potential conflict of interest.

## References

[1] Bhatia KP Bain P Bajaj N Elble RJ Hallett M Louis ED. Consensus statement on the classification of tremors. From the task force on tremor of the International Parkinson and Movement Disorder Society. Mov Disord. (2018) 33:75–87. 10.1002/mds.27121 29193359 PMC6530552

[2] Milanov I. Electromyographic differentiation of tremors. Clin Neurophysiol. (2001) 112:1626–32. 10.1016/s1388-2457(01)00629-0 11514245

[3] Hess CW Pullman SL. Tremor: clinical phenomenology and assessment techniques. Tremor Other Hyperkinet Mov. (2012) 2:1–15. 10.7916/D8WM1C41 23439931 PMC3517187

[4] Daube JR Rubin DI. Needle electromyography. Muscle Nerve. (2009) 39:244–70. 10.1002/mus.21180 19145648

[5] Grimaldi G Manto M. Neurological tremor: sensors, signal processing and emerging applications. Sensors (Basel). (2010) 10:1399–422. 10.3390/s100201399 22205874 PMC3244020

[6] Elble RJ McNames J. Using portable transducers to measure tremor severity. Tremor Other Hyperkinet Mov (NY). (2016) 6:375. 10.7916/D8DR2VCC 27257514 PMC4872171

[7] Heldman DA Jankovic J Vaillancourt DE Prodoehl J Elble RJ Giuffrida JP. Essential tremor quantification during activities of daily living. Parkinsonism Relat Disord. (2011) 17:537–42. 10.1016/j.parkreldis.2011.04.017 21570891 PMC3137659

[8] Dai H Zhang P Lueth TC. Quantitative assessment of Parkinsonian tremor based on an inertial measurement unit. Sensors (Basel). (2015) 15:25055–71. 10.3390/s151025055 26426020 PMC4634500

[9] Marino S Cartella E Donato N Muscarà N Sorbera C Cimino V. Quantitative assessment of Parkinsonian tremor by using biosensor device. Medicine (Baltimore). (2019) 98:e17897. 10.1097/MD.0000000000017897 31860947 PMC6940115

[10] Hssayeni MD Jimenez-Shahed J Burack MA Ghoraani B. Wearable sensors for estimation of Parkinsonian tremor severity during free body movements. Sensors (Basel). (2019) 19:4215. 10.3390/s19194215 31569335 PMC6806340

[11] Mahadevan N Demanuele C Zhang H Volfson D Ho B Erb MK. Development of digital biomarkers for resting tremor and bradykinesia using a wrist-worn wearable device. NPJ Digit Med. (2020) 3:5. 10.1038/s41746-019-0217-7 31970290 PMC6962225

[12] Dai H Cai G Lin Z Wang Z Ye Q. Validation of inertial sensing-based wearable device for tremor and bradykinesia quantification. IEEE J Biomed Health Inform. (2020) 25:997–1005. 10.1109/JBHI.2020.3009319 32750961

[13] Sanchez-Perez LA Sanchez-Fernandez LP Shaout A Martinez-Hernandez JM Alvarez-Noriega MJ. Rest tremor quantification based on fuzzy inference systems and wearable sensors. Int J Med Inform. (2018) 114:6–17. 10.1016/j.ijmedinf.2018.03.002 29673605

[14] Jeon H Lee W Park H Lee HJ Kim SK Kim HB. Automatic classification of tremor severity in Parkinson's disease using a wearable device. Sensors (Basel). (2017) 17:2067. 10.3390/s17092067 28891942 PMC5621347

[15] Rigas G Tzallas AT Tsipouras MG Bougia P Tripoliti EE Baga D. Assessment of tremor activity in the Parkinson's disease using a set of wearable sensors. IEEE Trans Inf Technol Biomed. (2012) 16:478–87. 10.1109/TITB.2011.2182616 22231198

[16] Charles SK Geiger DW Davidson AD Pigg AC Curtis CP Allen BC. Toward quantitative characterization of essential tremor for future tremor suppression. IEEE Int Conf Rehabil Robot. (2017) 2017:175–80. 10.1109/ICORR.2017.8009242 28813814

[17] Lonini L Dai A Shawen N Simuni T Poon C Shimanovich L. Wearable sensors for Parkinson's disease: which data are worth collecting for training symptom detection models. NPJ Digit Med. (2018) 1:64. 10.1038/s41746-018-0071-z 31304341 PMC6550186

[18] Huo W Angeles P Tai YF Pavese N Wilson S Hu MT. A heterogeneous sensing suite for multisymptom quantification of Parkinson's disease. IEEE Trans Neural Syst Rehabil Eng. (2020) 28:1397–406. 10.1109/TNSRE.2020.2978197 32305925

[19] Delrobaei M Memar S Pieterman M Stratton TW McIsaac K Jog M. Towards remote monitoring of Parkinson's disease tremor using wearable motion capture systems. J Neurol Sci. (2018) 384:38–45. 10.1016/j.jns.2017.11.004 29249375

[20] LeMoyne R Mastroianni T Cozza M Coroian C Grundfest W. Implementation of an iPhone for characterizing Parkinson's disease tremor through a wireless accelerometer application. In: Proceedings of the 32nd Annual International Conference of the IEEE Engineering in Medicine and Biology Society (EMBC). Buenos Aires (2010). p. 4954–8. 10.1109/IEMBS.2010.5627240 21096671

[21] LeMoyne R Tomycz N Mastroianni T McCandless C Cozza M Peduto D. Implementation of a smartphone wireless accelerometer platform for establishing deep brain stimulation treatment efficacy of essential tremor with machine learning. In: Proceedings of the 37th Annual International Conference of the IEEE Engineering in Medicine and Biology Society (EMBC). Milan (2015). p. 6772–5. 10.1109/EMBC.2015.7319948 26737848

[22] Araújo R Tábuas-Pereira M Almendra L Ribeiro J Arenga M Negrão L. Tremor frequency assessment by iPhone^®^ applications: correlation with EMG analysis. J Parkinsons Dis. (2016) 6:717–21. 10.3233/JPD-160936 27662333

[23] Bhatti D Thompson R Hellman A Penke C Bertoni JM Torres-Russotto D. Smartphone apps provide a simple, accurate bedside screening tool for orthostatic tremor. Mov Disord Clin Pract. (2017) 4:852–7. 10.1002/mdc3.12547 30363432 PMC6174519

[24] López-Blanco R Velasco MA Méndez-Guerrero A Romero JP del Castillo MD Serrano JP. Essential tremor quantification based on the combined use of a smartphone and a smartwatch: the NetMD study. J Neurosci Methods. (2018) 303:95–102. 10.1016/j.jneumeth.2018.02.015 29481820

[25] Varghese J Niewöhner S Soto-Rey I Schipmann-Miletić S Warneke N Warnecke T. A smart device system to identify new phenotypical characteristics in movement disorders. Front Neurol. (2019) 10:48. 10.3389/fneur.2019.00048 30761078 PMC6363699

[26] López-Blanco R Velasco MA Méndez-Guerrero A Romero JP del Castillo MD Serrano JI. Smartwatch for the analysis of rest tremor in patients with Parkinson's disease. J Neurol Sci. (2019) 401:37–42. 10.1016/j.jns.2019.04.011 31005763

[27] Shawen N O'Brien MK Venkatesan S Lonini L Simuni T Hamilton JL. Role of data measurement characteristics in the accurate detection of Parkinson's disease symptoms using wearable sensors. J Neuroeng Rehabil. (2020) 17:52. 10.1186/s12984-020-00684-4 32312287 PMC7168958

[28] Zajki-Zechmeister T Kögl M Kalsberger K Franthal S Homayoon N Katschnig-Winter P. Quantification of tremor severity with a mobile tremor pen. Heliyon. (2020) 6:e04702. 10.1016/j.heliyon.2020.e04702 32904326 PMC7452531

[29] Cole BT Roy SH De Luca CJ Nawab SH. Dynamical learning and tracking of tremor and dyskinesia from wearable sensors. IEEE Trans Neural Syst Rehabil Eng. (2014) 22:982–91. 10.1109/TNSRE.2014.2310904 24760943

[30] Kim J Parnell C Wichmann T DeWeerth SP. Longitudinal wearable tremor measurement system with activity recognition algorithms for upper limb tremor. Annu Int Conf IEEE Eng Med Biol Soc. (2016) 2016:6166–9. 10.1109/EMBC.2016.7592136 28269660

[31] Battista L Romaniello A. A novel device for continuous monitoring of tremor and other motor symptoms. Neurol Sci. (2018) 39:1333–43. 10.1007/s10072-018-3414-2 29736737

[32] Battista L Romaniello A. A wearable tool for selective and continuous monitoring of tremor and dyskinesia in Parkinsonian patients. Parkinsonism Relat Disord. (2020) 77:43–7. 10.1016/j.parkreldis.2020.06.020 32619969

[33] Heijmans M Habets J Kuijf M Kubben P Herff C. Evaluation of Parkinson's disease at home: predicting tremor from wearable sensors. Annu Int Conf IEEE Eng Med Biol Soc. (2019) 2019:584–7. 10.1109/EMBC.2019.8857717 31945966

[34] San-Segundo R Zhang A Cebulla A Panev S Tabor G Stebbins K. Parkinson's disease tremor detection in the wild using wearable accelerometers. Sensors (Basel). (2020) 20:5817. 10.3390/s20205817 33066691 PMC7602495

[35] McNames J Shah VV Mancini M Curtze C El-Gohary M Aboy M. A two-stage tremor detection algorithm for wearable inertial sensors during normal daily activities. Annu Int Conf IEEE Eng Med Biol Soc. (2019) 2019:2535–8. 10.1109/EMBC.2019.8857133 31946413

[36] Kuosmanen E Wolling F Vega J Kan V Nishiyama Y Harper S. Smartphone-based monitoring of parkinson disease: quasi-experimental study to quantify hand tremor severity and medication effectiveness. JMIR Mhealth Uhealth. (2020) 8:e21543. 10.2196/21543 33242017 PMC7728543

[37] Erb MK Karlin DR Ho BK Thomas KC Parisi F Vergara-Diaz GP. mHealth and wearable technology should replace motor diaries to track motor fluctuations in Parkinson's disease. NPJ Digit Med. (2020) 3:6. 10.1038/s41746-019-0214-x 31970291 PMC6969057

[38] Vescio B Nisticò R Augimeri A Quattrone A Crasà M Quattrone A. Development and validation of a new wearable mobile device for the automated detection of resting tremor in Parkinson's disease and essential tremor. Diagnostics (Basel). (2021) 11:200. 10.3390/diagnostics11020200 33573076 PMC7911899

[39] Hossen A Muthuraman M Al-Hakim Z Raethjen J Deuschl G Heute U. Discrimination of Parkinsonian tremor from essential tremor using statistical signal characterization of the spectrum of accelerometer signal. Biomed Mater Eng. (2013) 23:513–31. 10.3233/BME-130773 24165554

[40] Ghassemi NH Marxreiter F Pasluosta CF Kugler P Schlachetzki J Schramm A. Combined accelerometer and EMG analysis to differentiate essential tremor from Parkinson's disease. Annu Int Conf IEEE Eng Med Biol Soc. (2016) 2016:672–5. 10.1109/EMBC.2016.7590791 28268417

[41] Di Biase L Brittain JS Shah SA Pedrosa DJ Cagnan H Mathy A. Tremor stability index: a new tool for differential diagnosis in tremor syndromes. Brain. (2017) 140:1977–86. 10.1093/brain/awx104 28459950 PMC5493195

[42] Bove F Di Lazzaro G Mulas D Cocciolillo F Di Giuda D Bentivoglio AR. A role for accelerometry in the differential diagnosis of tremor syndromes. Funct Neurol. (2018) 33:45–9. 10.11138/fneur/2018.33.1.045 29633696 PMC5901940

[43] Iosa M Picerno P Paolucci S Morone G. Wearable inertial sensors for human movement analysis. Expert Rev Med Devices. (2016) 13: 641–59. 10.1080/17434440.2016.1198694 27309490

[44] Rovini E Maremmani C Cavallo F. How wearable sensors can support parkinson's disease diagnosis and treatment: a systematic review. Front Neurosci. (2017) 11:555. 10.3389/fnins.2017.00555 29056899 PMC5635326

[45] Monje MHG Foffani G Obeso J Sánchez-Ferro Á. New sensor and wearable technologies to aid in the diagnosis and treatment monitoring of Parkinson's disease. Annu Rev Biomed Eng. (2019) 21:111–43. 10.1146/annurev-bioeng-062117-121036 31167102

[46] Lu R Xu Y Li X Fan Y Zeng W Tan Y Ren K Chen W Cao X. Evaluation of wearable sensor devices in Parkinson's disease: a review of current status and future prospects. Parkinsons Dis. (2020) 2020:4693019. 10.1155/2020/4693019 33029343 PMC7530475

[47] Kägi G Bhatia KP Tolosa E. The role of DAT-SPECT in movement disorders. J Neurol Neurosurg Psychiatry. (2010) 81:5–12. 10.1136/jnnp.2008.157370 20019219

[48] Nisticò R Pirritano D Salsone M Novellino F Del Giudice F Morelli M. Synchronous pattern distinguishes resting tremor associated with essential tremor from rest tremor of Parkinson's disease. Parkinsonism Relat Disord. (2011) 17:30–3. 10.1016/j.parkreldis.2010.10.006 21071257

[49] Nisticò R Fratto A Vescio B Arabia G Sciacca G Morelli M. Tremor pattern differentiates drug-induced resting tremor from Parkinson disease. Parkinsonism Relat Disord. (2016) 25:100–3. 10.1016/j.parkreldis.2016.02.002 26895708

